# Influence of antihypertensive medications on the clinical parameters of anodized dental implants: a retrospective cohort study

**DOI:** 10.1186/s40729-020-00231-9

**Published:** 2020-07-20

**Authors:** Keisuke Seki, Akira Hasuike, Yoshihiro Iwano, Yoshiyuki Hagiwara

**Affiliations:** 1 Implant Dentistry, Nihon University School of Dentistry Dental Hospital, Tokyo, Japan; 2 Department of Comprehensive Dentistry and Clinical Education, Nihon University School of Dentistry, Tokyo, Japan; 3 Department of Periodontology, Nihon University School of Dentistry, Tokyo, Japan; 4 Private Practice, Tokyo, Japan

**Keywords:** Dental implants, Antihypertensive medications, Peri-implantitis, Clinical study

## Abstract

**Background:**

Hypertension is a chronic medical condition in which blood pressure in the arteries is elevated. Given the large proportion of dental implant patients using antihypertensive medications, it is crucial to evaluate the effects of these drugs on the clinical parameters of osseointegrated implants. The aim of the present retrospective cohort study was to evaluate the influence of antihypertensive medications on clinical peri-implant tissue parameters.

**Methods:**

Thirty-five patients received a total of 77 anodized dental implants. Based on the history of the use of antihypertensive medications, the patients were divided into two groups: the group taking antihypertensive medications (AH group) and the group of healthy patients (H group). Implants were followed up clinically and radiologically, with a focus on the peri-implant soft tissue parameters probing pocket depth, bleeding on probing, modified plaque index, and marginal peri-implant bone level stability.

**Results:**

None of the implants were lost, and no technical failures occurred. The mean follow-up duration was 7 years and 1 month. A significant difference was observed in the probing pocket depth 3.8 ± 1.3 mm in the AH group and 3.0 ± 0.7 mm in the H group. In the AH and H groups, 26.5% (9/34) and 4.7% (2/43) of the patients were diagnosed with peri-implantitis at the implant level, respectively.

**Conclusions:**

Our findings suggest some correlations between antihypertensive medication use and clinical parameters in anodized peri-implant tissue.

## Background

Dental implants are the best option for replacing missing teeth, which show sufficient longevity in most cases. Even though dental implants have a long-term success rate of more than 90%, some people experience complications as with any treatment modality. Technical and biological complications can occur in implant dentistry [1]. The biological complications related to dental implants include peri-implant mucositis and peri-implantitis. There is no definitive evidence regarding the etiology and clinical countermeasures for these two biological complications [2]. Various risk indicators have been discussed, and etiological similarities between periodontal and peri-implant tissues have been of interest to clinicians and researchers [3]. Both periodontitis and peri-implantitis are initiated by the accumulation of microbial biofilms on the hard surfaces of the teeth or dental implants [4, 5].

Researchers have recently shown considerable interest in possible links between periodontal disease and systemic diseases and conditions, such as diabetes mellitus, cardiovascular disease, bacterial pneumonia, and low birth weight [6]. Systemic diseases may impair the host’s barrier function and immune defense against periodontal pathogens, creating an opportunity for destructive periodontal disease and likely peri-implant diseases. Systemic diseases and medications may also interfere with healing in peri-implant sites and increase the risk of biological complications and implant failure [7].

In 2017, new guidelines from the American Heart Association, the American College of Cardiology, and nine other health organizations lowered the blood pressure criteria for the diagnosis of hypertension to 130/80 mmHg and higher for all adults [8]. The previous guidelines set the threshold at 140/90 mmHg for individuals younger than 65 years and 150/80 mmHg for those aged 65 years and older. It could then be estimated that 70 to 79% of men aged 55 years and older are classified as having hypertension.

The prevalence of hypertension is increasing in individuals older than 60 years in developed countries. Patients with hypertension also require dental implant therapy. Thus, the influence of antihypertensive medications on peri-implant tissue has been of great interest. Wu et al. conducted a retrospective cohort study on 728 patients that assessed the influence of antihypertensive medications on implant survival rates [9]. The study demonstrated a higher survival rate of osseointegration implants in patients prescribed with antihypertensive medications (hazard ratio, 0.12; 95% confidence interval [CI], 0.03–0.49). The authors explained this effect by the relationship between antihypertensive medication use and bone metabolism. This is the only study that has assessed the relationship between antihypertensive medication use and implant-related outcomes.

Peri-implant diseases should ideally be diagnosed in the early stages to prevent complications and achieve longevity. Although the implant survival rate would be the true endpoint for dental implant treatment, clinical surrogate endpoints are useful for detecting early onset of peri-implant disease [10]. For this purpose, it is necessary to further investigate the influence of antihypertensive medications on peri-implant clinical endpoints.

Biological peri-implant complications have a multifactorial etiology. Besides patient systemic factors, several other etiological factors for peri-implant complications also exist. It is well known that a myriad of possible risk factors could be associated with dental implant characteristics. Thus, to assess the impact of medications, patients who received implants with the same characteristics should be included in both the test and control groups. A recent systematic review showed lower failure rates for anodized implants than for turned implants as well as titanium plasma-sprayed, -blasted, and acid-etched implants [11]. The authors of this review explained that the oxidized surface provides a greater number of undercuts that may result in improved osseointegration.

In this retrospective cohort study, the clinical parameters in patients treated with anodized implants were evaluated to investigate the influence of antihypertensive medications on peri-implant tissues.

## Methods

### Patient selection and data sources

Ethical approval from the Nihon University School of Dentistry Ethics Committee (permit number: EP16D013) was obtained prior to initiating an exploratory analysis. Data of patients who provided written informed consent allowing the use of their records for research were used. The retrospective cohort included patients who had implants placed by a periodontist (K.S.) at Nihon University School of Dentistry Dental Hospital between June 2003 and May 2018. The inclusion criteria were as follows: (1) age more than 50 years on the day of dental placement, (2) two-stage dental implant placement with anodized implants, and (3) follow-up maintenance therapy at least 6 months after superstructure delivery. The exclusion criteria were as follows: (1) history of treatment for moderate or severe periodontitis, (2) smoking, (3) requirement for bone grafting before or at the time of implant placement, and (4) systemic disease other than hypertension.

**Fig. 1:**
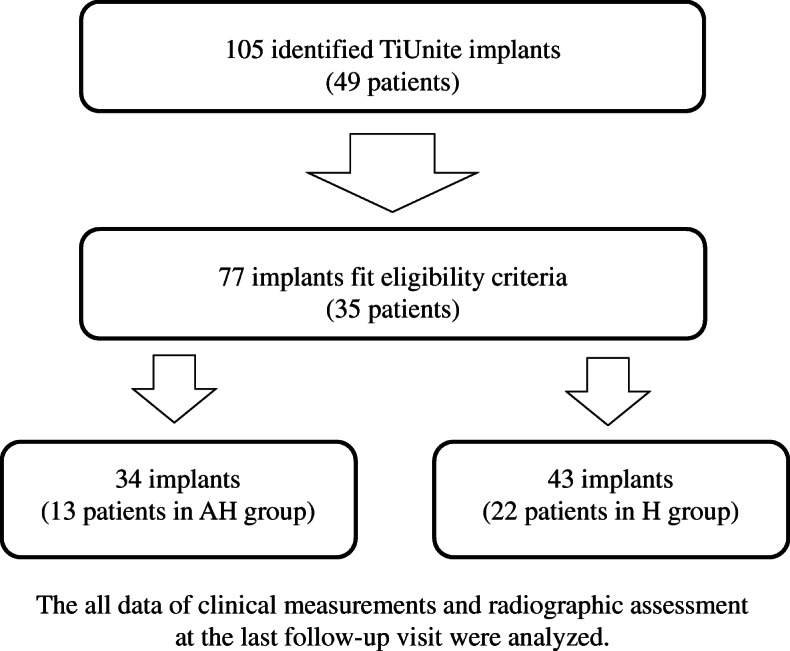
The all data of clinical measurements and radiographic assessment at the last follow-up visit were analyzed

We identified 105 implants inserted in 49 patients. We lost the follow-up dental records of 14 patients. Seventy-seven implants in 35 patients were divided into two groups based on the history of antihypertensive medication use. Calcium channel blocker (CCB) and angiotensin II receptor blocker (ARB) are two of the most widely used antihypertensive agents in Japan. These are often medicated in combination including other antihypertensive drugs. In particular, CCB/ARB combination therapy is more effective in lowering blood pressure than high-dose ARB therapy [12]. Hence, all antihypertensive medications used were investigated. Patients taking antihypertensive medications were assigned to the group taking antihypertensive medications for the treatment of hypertension (AH group), and patients not taking antihypertensive medications were assigned to the group with healthy patients (H group). The experimental procedure was exhibited in Fig. 1.

### Surgical protocol for implant placement

All implants were placed equally within the bone crest according to the manufacturer’s recommendations under local anesthesia by the periodontist (K.S.). All surgeries were enforced using a two-stage approach. None of the patients underwent bone grafting surgery. To prevent infection, we prescribed antibiotics (cefuroxime axetil, cefcapene pivoxil hydrochloride hydrate, and amoxicillin hydrate) to be taken orally three times a day for 3 days after either the primary or the secondary surgery. Painkillers (loxoprofen sodium hydrate and diclofenac sodium) were prescribed during pain episodes. Sutures were removed 1 week after surgery, and oral hygiene instructions were provided.

### Self-maintenance and the frequency of follow up visit

First, we evaluated a month after the superstructure was delivered. Then on after, the supportive periodontal and implant therapy was scheduled for follow-up appointments at 3-month intervals. Any patient with favorable hygiene conditions were checked every 6 months instead. For the areas of plaque accumulation, the patients were instructed the use of interdental brushes and enhanced her oral hygiene with regular professional cleanings. For professional tooth cleaning, a polishing paste not containing fluoride was used in regard for avoiding titanium corrosion.

### Clinical measurements

The subjects were clinically monitored at the last follow-up visit. The primary outcome of this cohort study was the probing pocket depth around the implant measured using a periodontal pocket probe (11 Colorvue® Probe Kit; Hu-Friedy, Chicago, IL, USA). For proper standardization, one examiner obtained all clinical measurements. The probing force (0.15 N) within individual standardization was employed using a precision scale to calibrate the force with repeated measurements. Subsequently, using the 6-point method (at the mesial and distal angles and at the center of the buccal and lingual aspects of the implant), we measured the probing pocket depth to the nearest millimeter at six sites per implant and calculated the average of the six points for each implant. Bleeding on probing (BoP) of the peri-implant mucosa was also assessed within 10 s after probing. The BoP criterion was as follows: negative (0, no bleeding) or positive (1, bleeding). The average of the six points was calculated, with the minimum BoP score detected at 0 and the maximum detected at 1. Plaque adhesion (modified Plaque Index: mPI) on the superstructure of the implant was measured on a 4-point scale (0, no plaque; 1, plaque seen with probing of the superstructure surface; 2, moderate accumulation of soft deposits visible with the naked eye; 3, abundance of soft deposit on the superstructure surface). The average score of four surfaces (buccal, lingual, mesial, and distal aspects) was calculated, with the minimum mPI score detected at 0 and the maximum detected at 3 [13].

### Radiographic assessment

Changes in bone loss around the implants over time were assessed by referring to digital radiographs obtained via the paralleling technique. All dental radiographies were irradiated at 110 kV, 1–20 mA, and an effective dose of 100 μSv. Bone loss was measured using images obtained from a digital radiograph viewing system (TechM@trix, SDS Viewer, TechMatrix Corporation, Tokyo, Japan). The vertical distances were measured from either the mesial or distal platform of the implant to the expected marginal bone level. The difference between the average value of mesial and distal bone losses was compared serially at baseline and follow-up examination. The measured serial data were evaluated for marginal bone loss. Informed consent was obtained from all patients, and the data were processed in such a manner that patient anonymity was ensured.

### Clinical diagnosis of peri-implant disease

During follow-up visits, implants that fulfilled the following criteria were diagnosed as having peri-implant mucositis or peri-implantitis. Peri-implant mucositis was defined as bleeding and/or suppuration at the implant site on probing without any signs of additive bone resorption after initial bone remodeling. Peri-implantitis was diagnosed when the probing pocket depth around the implant was 6 mm or more, when suppuration and bleeding were observed at the time of probing, and when bone resorption was present radiographically for 25% or more of the implant length [14].

### Statistical analysis

The statistical software GraphPad Prism 5.0 (GraphPad Software Inc., San Diego, CA, USA) was used to perform the data analysis. Image data were measured by the same examiner. Data from the AH and H groups were statistically compared using the Mann-Whitney *U* test (continuous variable) and chi-squared test (categorical variable). A multivariate analysis was performed using logistic regression analysis to examine potential effect of peri-implantitis. A level of 95% (*P* < 0.05) was considered statistically significant.

## Results

### Description of the study patients and implants

Seventy-seven implants (34 in the AH group and 43 in the H group) in 35 patients were included in this retrospective cohort study. Table 1 describes the study patients and implants. Twenty-one patients were women. Thirty-five implants were placed in the maxilla and 42 in the mandibular. The mean maintenance duration was 7 years and 1 month. The average patient age was 66.1 years. Thirteen patients with a combined 34 implants were taking an antihypertensive medication. The dental implants assessed in this study were the Nobel Replace^TM^ Select implant system (Nobel Biocare, Zürich, Switzerland), Nobel Replace^TM^ Groovy (Nobel Biocare), and Brånemark System® Mk III (Nobel Biocare). The patients were taking calcium antagonists, angiotensin II receptor blockers, and thiazide diuretics as either a monotherapy or a combined regimen (Table 2).

**Table 1:** Characteristics of the patients in the AH and H group

		AH group	H group	*P* value
**Total number (patients/implants)**		13/34	22/43	–
**Women (patients/implants)**		5/16	16/31	0.025*
**Age (mean), year**		66.7 ± 9.0	65.6 ± 10.0	ns
**Implant site (maxilla/mandibular)**		21/13	14/29	0.011*
**Mean maintenance duration**		6 years and 10 months	7 years and 3 months	ns
**Type of implants (patients/implants)**				
	**Nobel Replace**^**TM**^ **Select implant system**	3/8	5/11	
	**Nobel Replace**^**TM**^ **Groovy**	6/17	12/25	
	**Brånemark system® Mk III**	4/9	5/7	
**Antihypertensive medication (patients/implants: running number)**				
	**Calcium antagonists**	6/19	–	
	**Angiotensin II receptor blockers**	8/18	–	
	**Beta-blockers**	0/0	–	
	**Thiazide diuretics**	1/2	–	

*AH group* group taking antihypertensive medications, *H group* group with healthy patients*, ns* not significant

*P < 0.05

**Table 2:** Details of the patients in the AH group

Patient	Age	Site	Implant	Diseases	Maintenance	Antihypertensive medication
	(Sex)				(months)	
**1**	61	47	Brånemark Mk III Groovy	None	25	Angiotensin II receptor blockers
	(F)					
**2**	62	24, 27, 45, 47	Brånemark Mk III Groovy	None	35	Calcium antagonists
	(F)					
**3**	57	36, 37, 46	Brånemark Mk III Groovy	Peri-implant mucositis [46]	52	Calcium antagonists
	(M)			Peri-implantitis [37]		Angiotensin II receptor blockers
**4**	63	14, 15, 25	Replace Select Tapered	Peri-implantitis [15, 25]	116	Angiotensin II receptor blockers
	(F)					
**5**	55	46, 47	Replace Select Straight	Peri-implant mucositis [47]	108	Angiotensin II receptor blockers
	(M)					
**6**	77	24, 26, 27	Replace Select Tapered	Peri-implant mucositis [24]	165	Calcium antagonists
	(M)					
**7**	84	15, 16, 36, 46	Replace Groovy Straight	Peri-implantitis [15, 16]	153	Angiotensin II receptor blockers
	(M)					
**8**	67	16, 23, 25, 26, 36, 46	Replace Groovy Straight	Peri-implant mucositis [26]	132	Calcium antagonists
	(F)			Peri-implantitis [16, 23, 25, 46]		
**9**	64	25, 27	Replace Groovy Straight	None	37	Calcium antagonists
	(M)					
**10**	68	13, 15	Replace Groovy Straight	None	15	Angiotensin II receptor blockers
	(F)					
**11**	80	24	Replace Select Tapered	Peri-implant mucositis [24]	164	Calcium antagonists
	(M)					
**12**	63	15, 16	Replace Groovy Straight	Peri-implant mucositis [15]	27	Angiotensin II receptor blockers
	(M)					Thiazide diuretics
**13**	51	37	Brånemark Mk III Groovy	None	28	Angiotensin II receptor blockers
	(M)					

*AH group* group taking antihypertensive medications

### Clinical and radiographic parameters

The clinical and radiographic parameters are shown in Table 3. The probing pocket depth in the total study population was 3.4 ± 1.1 mm (mean ± standard deviation); a significant difference was observed between the AH group and H group, 3.8 ± 1.3 mm (median, 3.3 mm) and 3.0 ± 0.7 mm (median, 3.0 mm), respectively. The BoP score in the total study population was 0.2 ± 0.2, whereas the mean scores in the AH and H groups were 0.3 (median, 0.2) and 0.2 (median, 0.2), respectively. The mPI score in the total study population was 0.5 ± 0.7, whereas the mean scores in the AH and H groups were 0.7 (median, 0.5) and 0.4 (median, 0.0), respectively.

**Table 3:** Clinical and radiographic parameters

	AH group	H group	*P* value
**Probing pocket depth (mm)**	3.8 ± 1.3	3.0 ± 0.7	0.001^**^
**Bleeding on probing (score 0–1)**	0.3 ± 0.3	0.2 ± 0.2	ns
**Modfied plaque index (score 0–3)**	0.7 ± 0.8	0.4 ± 0.7	ns
**Marginal bone loss (mm)**	0.66 ± 1.17	0.34 ± 0.57	ns

*AH group* group taking antihypertensive medications, *H group* group with healthy patients*, ns* not significant

**P < 0.01

For the marginal bone loss in relation to the radiographic parameter, the mean follow-up duration was 84.8 months (range, 15–170 months; median, 82.0 months). The mean measurement in the total study population was 0.48 ± 0.89 mm, whereas the mean measurements in the AH and H groups were 0.66 mm (median, 0.40 mm; follow-up duration, 82.3 ± 53.3 months) and 0.34 mm (median, 0.30 mm; follow-up duration, 86.8 ± 48.8 months), respectively.

### Prevalence of peri-implant mucositis and peri-implantitis

Among all the subjects during the maintenance period, six patients (11 implants) were diagnosed with peri-implantitis (Table 4). In the AH group, 30.8% (4/13) and 26.5% (9/34) were diagnosed with peri-implantitis at the patient and implant levels, respectively; in the H group, 9.1% (2/22) and 4.7% (2/43) were diagnosed with such, respectively. Significant differences were confirmed only at the implant level, not at the patient level. The implants in the AH group were more susceptible to peri-implant diseases than those in the H group, with an odds ratio of 7.38 (95% CI, 1.474–36.954). Significant differences were not observed in the morbidity rates of peri-implant mucositis and peri-implant disease.

**Table 4:** Proportion of patients and implants diagnosed with peri-implant disease

	AH group	H group	*P* value	Odds ratio [95% CI]
	Patients (number/%)	Patients (number/%)		Patients
	Implants (number/%)	Implants (number/%)		Implants
**Peri-implant mucositis**	6/46.2%	7/31.8%	ns	–
	6/17.6%	9/20.9%	ns	–
**Peri-implantitis**	4/30.8%	2/9.1%	ns	–
	9/26.5%	2/4.7%	0.007^**^	7.38 [1.47–36.95]
**Peri-implant disease**	8/61.5%	9/40.9%	ns	–
	15/44.1%	11/25.6%	ns	–

*AH group* group taking antihypertensive medications, *H group* group with healthy patients, *CI* confidence interval, *ns* not significant

**P < 0.01

### A multivariate analysis (logistic regression analysis)

A multivariate analysis was performed using logistic regression analysis for peri-implantitis (Table 5). The peri-implantitis development was the objective variable, and the age, gender, maintenance duration, implant site, and the history of antihypertensive medications were used as the explanatory variable. Only the history of antihypertensive medications (OR = 12.90) showed a statistically significant result, whereas there was no significant difference in other explanatory valuables.

**Table 5:** Logistic regression analysis for peri-implantitis as the objective variable

Explanatory variable	Odds ratio [95% CI]	*P* value
Age (year)	1.11 [0.97–1.27]	0.128
Gender (man; 0, woman; 1)	8.71 [0.86–88.60]	0.667
Maintenance duration (month)	1.00 [0.99–1.02]	0.646
Implant site (mandibular; 0, maxilla; 1)	4.02 [0.68–23.60]	0.124
Antihypertensive medication (yes; 0, no; 1)	12.90 [1.60–104.00]	0.016*
*R*^2^=0.35		

*CI* confidence interval

*P < 0.05

## Discussion

In the present study, a higher prevalence of deep pockets was confirmed in the TiUnite implants placed in the patients prescribed with antihypertensive medications. Furthermore, a high prevalence of peri-implantitis was also observed at the implant level in the patients taking antihypertensive medications.

Based on the concept of periodontal medicine, periodontal disease might be a risk indicator for several systemic conditions, such as diabetes mellitus and coronary heart disease (CHD) [15]. In previous epidemiological studies, associations between CHD and periodontal diseases have been described [16]. In contrast, reports mentioning the relationship between CHD and peri-implant diseases are limited. Researchers have failed to demonstrate that systemic disorder-related factors (i.e., smoking and high blood pressure) are definitive risk factors for developing peri-implantitis [17, 18]. However, it is also true that the proportion of patients with peri-implantitis with diabetes mellitus and hypertension is high [19]. Because no definitive opinion on the relationship between peri-implant inflammation and hypertension exists, the present study findings are quite valuable.

In the present study, the mean age of the participants was 66.1 years, and the mean age of the AH group patients was 66.7 years, which indicates the accordance of patients’ age in relation to the presence of hypertension and use of dental implants. The AH group used various antihypertensive medication combinations, and several patients took multiple medications, which reflects the current medical situation among Japanese patients with hypertension. The average follow-up duration was approximately 7 years. Because 5 or more years of observation are needed to assess the stability of tissue around dental implants [4], the average follow-up duration of the present study was long enough. The proportions of the patients with peri-implantitis were 17.1% and 14.3% at the patient and implant levels, respectively. Although the proportions were relatively small compared with those in previous reports, considering the narrow inclusion criteria (excluding smokers and patients with periodontal disease), the proportions were not very small.

For the probing pocket depth, a significant difference was observed between the AH and H groups. This result indicates possible medication-induced gingival hyperplasia in patients with hypertension, which is known to be a side effect of calcium channel blocking agents [20]. It is known that the symptoms begin with cation flux inhibition, which leads to decreased cellular folate uptake, changes in matrix metalloproteinase metabolism, and failure to activate collagenase. Decreased availability of activated collagenase results in decreased degradation of accumulated connective tissue. To date, no reports of hyperplasia in peri-implant tissue are available; however, the results of the present study suggest some correlations. There are several differences between periodontal and peri-implant tissues, such as the presence of the periodontal ligament, orientation of collagen fibers, and properties of exudate. Considering these differences, further assessment regarding hyperplasia in peri-implant tissue is needed. Furthermore, other antihypertensive medications, such as angiotensin receptor blockers and thiazide diuretics, were also included in this study. Assessment of the influence of these medications on peri-implant tissue is needed.

There was no significant difference in bleeding on probing and modified plaque index parameters between the two groups. However, both values tended to be larger in the AH group. Hence, it was suggested that the hygiene around the implant was poor and there was inflammatory response compared to the H group.

Although there was a significant difference in the probing pocket depth, the marginal bone loss in relation to the radiographic parameter was quite small in both groups. This could be attributable to the influence of both antihypertensive medications and the surface characteristics of TiUnite implants. Generally, antihypertensive medications are known to affect bone metabolism. Recent animal studies involving antihypertensive medications and implants have shown that the bone implant contact percentage and peri-implant bone tissue volume of rats are significantly increased by propranolol, a non-selective beta-blocker [21]. In a study of spontaneously hypertensive rats, it was observed that the expression of bone resorption markers was decreased, and that the cortical levels of TRAP+ cells were increased in the group taking calcium channel blockers [22]. In clinical studies of humans, favorable results were observed in the group taking calcium channel blockers based on the findings of dental cone beam computed tomography, which was used to evaluate the maxillary bone density of patients with hypertension who were administered with chronic antihypertensive medications [23]. In a cohort study on the survival rate of dental implants with antihypertensive therapy, it was concluded that the implant survival rate in antihypertensive medication users was higher than that in non-users [9]. On the other hand, survival rate was not examined in this study. Our results were exhibited increasing of probing depth and peri-implantitis prevalence in AH group, which means the implant success rate has worsened. Thus, it was suspected that the implant success rate stays low during maintenance period even if not causing implant loss under the antihypertensive medications. However, no articles in this area have been reported, and future research is needed.

The results of multivariate analysis in our study revealed that the antihypertensive medication affects the development of peri-implantitis. We found that the adjusted data for multivariate analysis were higher than the crude data in history of antihypertensive medications. In terms of eliminating confounding factors, this finding suggests that the medications might be a risk factor for the outcome of peri-implantitis. In the future, we need to be careful hearing history of antihypertensive medications in order to achieve implant maintenance successfully.

One of the limitations of the present study was the small sample size. Only nonsmoker patients without periodontal and systemic diseases other than hypertension were included in this study, and the strict inclusion criteria decreased the sample size. In the future, a prospective observational study with a large sample size on individual medications and statistical adjustment of covariates should be conducted.

## Conclusions

We examined the clinical parameters in patients who received long-term implant maintenance. There were significant differences between the AH and H groups in terms of the probing pocket depth and proportion of implants diagnosed as having peri-implantitis. Our findings suggest some correlations between antihypertensive medication use and clinical parameters in anodized peri-implant tissue.

## Data Availability

The datasets used and analyzed during the current study are available from the corresponding author upon reasonable request.

## Abbreviations

CI: Confidence interval
BoP: Bleeding on probing
CHD: Coronary heart disease

## References

[1] Heitz-Mayfield LJ, Needleman I, Salvi GE, Pjetursson BE (2014). Consensus statements and clinical recommendations for prevention and management of biologic and technical implant complications. Analysis of risk factors for cluster behavior of dental implant failures. Int J Oral Maxillofac Implants..

[2] Hasuike A, Ueno D, Nagashima H, Kubota T, Tsukune N, Watanabe N (2019). Methodological quality and risk-of-bias assessments in systematic reviews of treatments for peri-implantitis. J Periodontal Res..

[3] Carcuac O, Berglundh T (2014). Composition of human peri-implantitis and periodontitis lesions. J Dent Res..

[4] Heitz-Mayfield LJ (2008). Peri-implant diseases: diagnosis and risk indicators. J Clin Periodontol..

[5] Ogata Y, Nakayama Y, Tatsumi J, Kubota T, Sato S, Nishida T (2017). Prevalence and risk factors for peri-implant diseases in Japanese adult dental patients. J Oral Sci..

[6] Falcao A, Bullón P (2019). A review of the influence of periodontal treatment in systemic diseases. Periodontol 2000.

[7] Turri A, Rossetti PH, Canullo L, Grusovin MG, Dahlin C (2016). Prevalence of peri-implantitis in medically compromised patients and smokers: a systematic review. Int J Oral Maxillofac Implants..

[8] Whelton PK, Carey RM (2018). The 2017 American College of Cardiology/American Heart Association clinical practice guideline for high blood pressure in adults. JAMA Cardiol..

[9] Wu X, Al-Abedalla K, Eimar H (2016). Antihypertensive medications and the survival rate of osseointegrated dental implants: a cohort study. Clin Implant Dent Relat Res..

[10] Renvert S, Persson GR, Pirih FQ, Camargo PM (2018). Peri-implant health, peri-implant mucositis, and peri-implantitis: case definitions and diagnostic considerations. J Periodontol..

[11] Wennerberg A, Albrektsson T, Chrcanovic B (2018). Long-term clinical outcome of implants with different surface modifications. Eur J Oral Implantol..

[12] Miura S, Saku K (2012). Efficacy and safety of angiotensin II type 1 receptor blocker/calcium channel blocker combination therapy for hypertension: focus on a single-pill fixed-dose combination of valsartan and amlodipine. J Int Med Res..

[13] Seki K, Nakabayashi S, Tanabe N, Kamimoto A, Hagiwara Y (2017). Correlations between clinical parameters in implant maintenance patients: analysis among healthy and history-of-periodontitis groups. Int J Implant Dent..

[14] Berglundh T, Armitage G, Araujo MG, Avila-Ortiz G, Blanco J, Camargo PM (2018). Peri-implant diseases and conditions: consensus report of workgroup 4 of the 2017 World Workshop on the Classification of Periodontal and Peri-Implant Diseases and Conditions. J Periodontol..

[15] Beck JD, Moss KL, Morelli T, Offenbacher S (2018). Periodontal profile class is associated with prevalent diabetes, coronary heart disease, stroke, and systemic markers of C-reactive protein and interleukin-6. J Periodontol..

[16] Beukers NG, van der Heijden GJ, van Wijk AJ, Loos BG (2017). Periodontitis is an independent risk indicator for atherosclerotic cardiovascular diseases among 60 174 participants in a large dental school in the Netherlands. J Epidemiol Community Health..

[17] Moy PK, Medina D, Shetty V, Aghaloo TL (2005). Dental implant failure rates and associated risk factors. Int J Oral Maxillofac Implants..

[18] Alsaadi G, Quirynen M, Komárek A, van Steenberghe D (2008). Impact of local and systemic factors on the incidence of late oral implant loss. Clin Oral Implant Res..

[19] Altay MA, Tozoglu S, Yıldırımyan N, Özarslan MM (2018). Is history of periodontitis a risk factor for peri-implant disease? A pilot study. Int J Oral Maxillofac Implants..

[20] Seymour RA, Ellis JS, Thomason JM (2000). Risk factors for drug-induced gingival overgrowth. J Clin Periodontol..

[21] Al-Subaie AE, Laurenti M, Abdallah MN, Tamimi I, Yaghoubi F, Eimar H (2016). Propranolol enhances bone healing and implant osseointegration in rats tibiae. J Clin Periodontol..

[22] Bastos MF, de Araújo IV, Brilhante FV, Pires AG, Goncalves TE, Napimoga MH (2013). Effects of lercanidipine on bone density and bone repair in spontaneously hypertensive rats. Implant Dent..

[23] Ağaçayack KS, Güven S, Koparal M, Güneş N, Atalay Y, Atilgans S (2014). Long-term effects of antihypertensive medications on bone mineral density in men older than 55 years. Clin Interv Aging..

